# Parental knowledge and access barriers in the management of cleft lip and/or palate: A cross-sectional study from Saudi Arabia

**DOI:** 10.1097/MD.0000000000046627

**Published:** 2026-01-16

**Authors:** Sari M. Rabah, Heba Jafar Sabbagh, Alya AlZabin, Ebtesam Almajed, Razan Albrahim, Reema Aldawish, Lara Alyahiwi, Rand Alshabnan, Narmin Mohammedsaeed Helal

**Affiliations:** a Plastic and Reconstructive Surgery Division, Department of Surgery, King Abdullah bin Abdulaziz University Hospital, Princess Nourah bint Abdulrahman University, Riyadh, Saudi Arabia; b Pediatric Dentistry Department, Faculty of Dentistry, King Abdulaziz University, Jeddah, Saudi Arabia; c College of Medicine, Princess Nourah bint Abdulrahman University, Riyadh, Saudi Arabia.

**Keywords:** attitude toward cleft lip and palate, awareness, care barriers, cleft lip, cleft palate, craniofacial abnormality, healthcare access, knowledge, misconceptions, Saudi Arabia

## Abstract

Cleft lip and/or cleft palate (CLP) are congenital anomalies caused by improper fusion of facial structures, influenced by genetic, environmental, and nutritional factors. Understanding parental knowledge and attitudes is essential, as misconceptions and limited awareness can affect the care and development of affected children. This study aimed to comprehensively evaluate Saudi parents’ understanding, perspectives, and behaviors concerning CLP in their children. Additionally, it sought to identify prevalent misconceptions and obstacles encountered by these parents in accessing necessary care. A cross-sectional survey was administered to parents of children with CLP in Saudi Arabia. The study utilized hospital databases, social media, and community centers to recruit participants, and collected sociodemographic details, knowledge, attitudes, practices, and care access barriers related to their children’s condition. A total of 156 Saudi parents participated in the study. Most (91%) recognized the need for multidisciplinary care, and 98.1% identified surgery as the main treatment, but only 14.1% were aware of syndromic associations, and 12.8% believed CLP could be prevented. Higher knowledge scores were significantly associated with socioeconomic status (r = .433, *P* < .01) and proper feeding positions (B = 1.20, *P* = .010). The findings indicate that while Saudi parents possess adequate knowledge of surgical and multidisciplinary management of cleft conditions, gaps persist in awareness of preventive measures and early intervention. Socioeconomic disparities and limited healthcare access highlight the need for enhanced educational initiatives and improved support services.

## 1. Introduction

Cleft lip and cleft palate (CLP) are serious congenital anomalies caused by the failure of facial structures to fuse properly during early embryonic development. These conditions result in openings in the upper lip, alveolus, or palate, and their severity depends on the timing and location of the developmental disruption.^[1]^ The exact cause of CLP is often unclear, but it is believed to result from a combination of genetic factors, environmental influences, and nutritional deficiencies.^[2]^ Children with CLP often experience maxillary hypoplasia and oral breathing, which can lead to reduced saliva production and increase the risk of periodontal diseases and oral disorders.^[3]^ Results from a study indicate that children with oral clefts exhibit higher healthcare utilization compared to their siblings without clefts, with congenital malformation-related hospital stays and medication intake being the primary contributing factors.^[4]^ Early therapies like lip taping and Nasoalveolar Molding (NAM) are frequently used during the newborn period to lessen the severity of cleft abnormalities.^[5]^ Gaining insight into how families and communities perceive CLP is crucial, as it highlights key aspects of parents’ emotional journeys, initial reactions, adaptation processes, and their experiences with professional and social support, all of which are essential for fostering the overall development of affected children.^[6,7]^ Many parents of children with CLP held misconceptions about its causes. They had a limited understanding of treatment timelines and the roles of care team members, underscoring the need for better parental education and support.^[8]^

Upon reviewing the literature, it was determined that no studies have been conducted in Saudi Arabia concerning parental knowledge of CLP. As parents play a significant role in children’s social and emotional development, and because there is limited information about their level of awareness and knowledge concerning CLP, the present study aimed to assess the knowledge, attitude, practice, misconceptions, and care barriers of Saudi parents of affected children with CLP.

## 2. Materials and methods

The study was conducted according to the guidelines of the Declaration of Helsinki and approved by the Institutional Review Board at Princess Nourah bint Abdulrahman University (PNU) (IRB log number: 22-1166). All participants were provided with informed consent prior to the commencement of the study. The research objectives were explained to all participants, and it was clarified that they could withdraw from the study without any consequences.

### 2.1. Sample and data collection

This cross-sectional study was conducted in Saudi Arabia between June 2023 and June 2024. Participants, consisting of children with CLP and their parents, were recruited via hospital records, social media platforms, and community support centers. Eligible participants were adults aged 18 or older who were the biological or legal guardians of a child diagnosed with CLP and had given informed consent. Those under 18, parents of children without a CLP diagnosis, or individuals who did not consent or later withdrew their consent were excluded from the study. Sample size calculation was conducted using G*Power software (Version 3.1.9.6), according to Linear multiple regression, effect size 0.15, when the level of confidence is 95% (alpha = 0.05), margin of error of 5%, power of 80%, and 3 total number of predictors, the total representative sample size required is 68.

### 2.2. Study survey

Data was collected using a structured questionnaire with a total of 44 questions distributed into 7 sections. The research team created sections 1, 2, and 5 of the questionnaire and subsequently reviewed and validated them by 2 medical professionals with expertise in the relevant field. Sociodemographic information: Includes 7 items covering the ages of the parents and child, place of residence, educational levels of the parents, and household income. CLP Description: Contains 6 questions regarding the child’s cleft condition, including cleft type, family history, diagnosis timing, relationship to the child, and any related anomalies. Knowledge of CLP: Consists of 9 questions evaluating general understanding of CLP, including its definition, diagnosis, prognosis, syndromic associations, risk factors, and sources of information. Most responses are in a “Yes,” “No,” or “I do not know” format. Questions 1 and 4 of section 3 were drawn from a validated questionnaire,^[9]^ and question 9 was derived from a validated questionnaire.^[10]^ The authors developed any remaining questions. Management of CLP: Includes 7 questions examining knowledge of CLP treatment, involved specialists, treatment stages, potential complications, and options for surgical or prosthetic management. Question 2 of section 4 of the study was adapted from a validated questionnaire,^[10]^ with the rest developed by the authors. Practices Toward CLCP: Consists of 5 questions assessing parental practices, including the timing of medical consultation, feeding methods, use of special formulas or feeding devices, and feeding positions. Attitudes Toward CLP: Contains 1 question assessing general attitudes, drawn from a validated tool.^[11]^ Barriers to care: Evaluates obstacles to healthcare access across 3 domains: geographic accessibility (3 questions), appointment availability and ease of access (4 questions), and scheduling-related issues (2 questions). This section is based on a validated questionnaire used to assess caregiver challenges in accessing care.^[12]^

### 2.3. Statistical analysis

The descriptive analysis, including the mean and standard deviation, was applied to continuous variables, while frequencies and percentages were used for categorically measured variables. The multiple response dichotomies analysis was used to describe the variables measured with more than 1 option. The Kolmogorov-Smirnov statistical test of Normality was used to assess the normality assumption for metric variables, along with the histograms. The Cronbach alpha test was used to test the internal consistency and reliability of the measured questionnaires. The chi-squared test of independence was used to assess the correlations between categorically measured variables, and Pearson correlation test was applied to assess the correlations between metric variables. However, the Likelihood Ratio adjusted chi-squared test of association was also preferred where necessary. The categorical factor analysis was used as a dimension reduction technique to compute the parental Households socioeconomic state (SES) index from their measured income sufficiency level, educational levels and residential regions, also the explorative factor analysis was used to assess the dimensionality of the parental perceptions about the access to medical appointments and difficulties to the medical services, treating physicians and missed medical appointments in order to reduce these multiple measured perceptions into smaller set of meaningful but simpler concepts that can be analyzed with multivariable regression analysis methods. The Multivariable Generalized Linear Regression Analysis (GLM) with Gamma was applied to assess the statistically significant predictors for parental perceptions of mean perceived missed medical appointments and travel difficulties to the medical facilities, the association between independent predictors variables with the analyzed outcome was expressed as multivariable adjusted risk rates with its 95% confidence intervals, the Gamma distribution regression was preferred over conventional regression methods because of the presence of skewed multivariable residuals and over-dispersion in model residuals.

Additionally, the multivariable linear regression (MLRA) analysis test was applied to assess parents’ overall mean knowledge of CLP and their perceived difficulty in accessing CLP treatment by physicians. This analysis regressed those scores against individuals’ sociodemographic factors, other child disease-related factors, and characteristics. The associations between people’s predictors and the analyzed outcomes were expressed as beta coefficients with their associated 95% confidence intervals. The SPSS IBM statistical computing program version 28 was used for the statistical data analysis, and the alpha significance value was considered at the 0.050 level.

## 3. Results

156 Saudi Arabia resident parents of CLP-affected children were enrolled in the study. Table 1 provides an overview of the sociodemographic characteristics of the participants. On average, the children were just over 4 years old (M = 4.04, SD = 3.83), with nearly half (45.5%) falling in the 1–3 years age range. Infants under 1 year made up 17.3% of the sample, while smaller proportions of children were aged 4–6 years (16.7%), 7–10 years (9.6%), and 11 years or older (10.9%). Mothers had a mean age of 34 years (SD = 7.33), and fathers were, on average, slightly older at 39.10 years (SD = 7.74). In terms of education, more than half of the mothers (54.5%) and just over half of the fathers (51.3%) held university degrees. Around a third of both mothers and fathers had completed high school or less (37.2%), and a smaller group reported holding postgraduate qualifications (mothers: 8.3%; fathers: 11.5%). Household income levels varied, but the majority of families (51.9%) said they could meet both routine and emergency expenses. Another 23.7% were able to meet only their routine expenses, while 16.7% reported they could save or invest. A small group (7.7%) indicated they were currently in-debt.

**Table 1:** Sociodemographic characteristics, N = 156.

Affected child age (years), mean (SD)	Frequency	Percentage
Child age group		4.04 (3.83)
<1 yr	27	17.3
1–3 yr	71	45.5
4–6 yr	26	16.7
7–10 yr	15	9.6
≥11 yr	17	10.9
Mother’s age (yr), mean (SD)		34 (7.33)
Father’s age (yr), mean (SD)		39.10 (7.74)
Mother’s educational level		
High school or less	58	37.2
University degree	85	54.5
Postgraduate degree	13	8.3
Father’s educational level		
High school or less	58	37.2
University degree	80	51.3
Postgraduate degree	18	11.5
Households’ monthly income sufficiency		
In-debt	12	7.7
Just meet routine expenses	37	23.7
Meet routine expenses and emergency expenses	81	51.9
Able to save/invest money	26	16.7

Overall, most families in the study had at least a moderate level of education and financial security, which could play an important role in shaping their knowledge, attitudes, and access to care for CLP-related health services.

**Figure 1.**
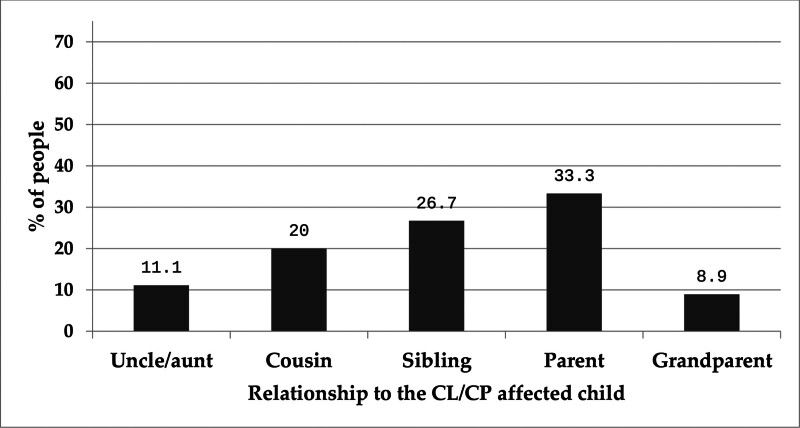
The family members who are CL/CP affected, besides the affected child. CL/CP = cleft lip and/or cleft palate.

**Figure 2.**
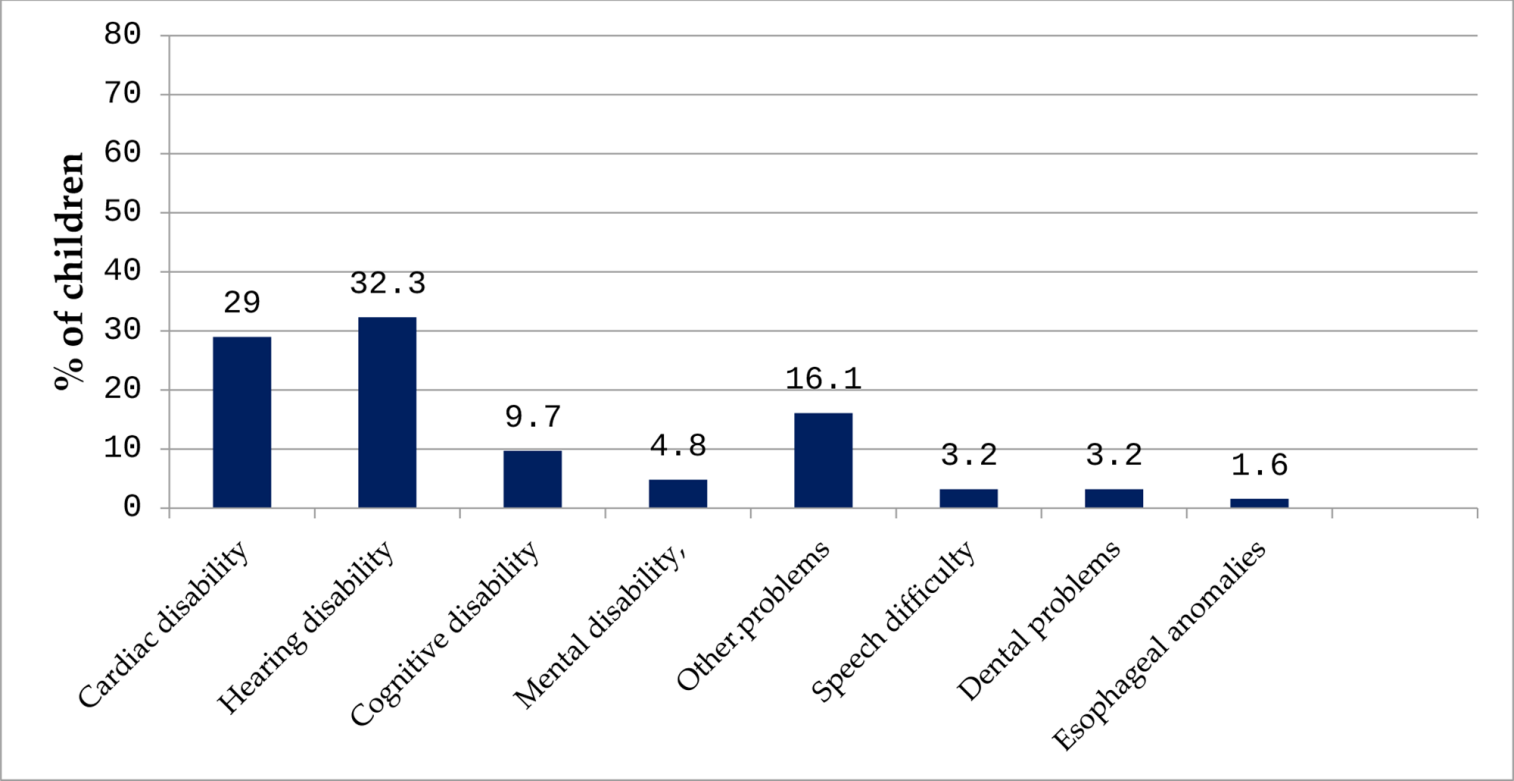
The CL/CP children associated with other congenital anomalies. CL/CP = cleft lip and/or cleft palate.

Table 2 presents a detailed overview of the medical and family history of children diagnosed with CLP. Nearly 1-third of families (28.8%) reported having another family member affected by CLP, most commonly a parent (33.3%), sibling (26.7%), or cousin (20%). Less frequent relations included uncles/aunts (11.1%) and grandparents (8.9%), indicating a possible hereditary component in a significant proportion of cases (Fig. 1). In terms of anatomical involvement, cleft types varied between the right and left sides of the lip. On the right side, 43.6% of children were unaffected, 21.2% had an incomplete cleft, and 35.3% had a complete right cleft. On the left side, 45.9% were unaffected, while 22.6% had an incomplete cleft and 31.5% had a complete cleft. Regarding the palate, a majority of children (55.1%) had a complete cleft palate, 30.8% had an incomplete cleft, and only 14.1% were unaffected. Most cleft cases were not diagnosed during pregnancy (71.8%), though just over a quarter (28.2%) were identified prenatally, suggesting room for improvement in early detection practices. About 1-quarter of the children (25.6%) had associated anomalies, while 65.4% did not, and 9% of parents were unsure. Among the 129 children with known anomalies, hearing disabilities (32.3%) and congenital cardiac conditions (29%) were most common. Other reported anomalies included cognitive disabilities (9.7%), other unspecified problems (16.1%), mental disabilities (4.8%), speech and dental issues (both 3.2%), and esophageal anomalies (1.6%) (Fig. 2). When asked about sources of information on CLP (n = 500), parents most frequently cited hospitals (46.2%), followed by the internet (29.5%) and social media (19.9%). Far fewer parents relied on family members (5.8%) or casual conversations (0.6%). Interestingly, knowledge of CLP was almost evenly divided: 49.4% of parents first learned about the condition during pregnancy, while 50.6% only became aware after having an affected child. Table 3 outlines parental knowledge regarding various aspects of CLP, revealing mixed levels of understanding across different areas. Two-thirds of respondents (66%) correctly identified a cleft as an opening on both the lips and hard palate, while smaller groups gave partially correct or incorrect responses. A notable knowledge gap was evident around the syndromic nature of CLP, with only 14.1% correctly recognizing that clefts can be part of a syndrome, while 44.2% admitted they did not know. Awareness of associated anomalies was somewhat stronger, with 64.1% correctly acknowledging that CLP may occur alongside other health conditions. Similarly, 74.4% knew that CLP can be diagnosed prenatally. However, only 12.8% believed CLP could be prevented, highlighting a limited understanding of modifiable risk factors like maternal health and prenatal exposures.

**Table 2:** Descriptive analysis of the children’s CLP medical history.

	Frequency	Percentage
Do you have any other family members with CLP?		
No	111	71.2
Yes	45	28.8
What is the relationship of this CLP-affected person to the child?		
Uncle/aunt	5	11.1
Cousin	9	20
Sibling	12	26.7
Parent	15	33.3
Grandparent	4	8.9
Child’s right side CLP type		
Not affected	68	43.6
Incomplete	33	21.2
Complete	55	35.3
Child’s left side CLP type		
Not affected	232	45.9
Incomplete	114	22.6
Complete	159	31.5
What type of cleft palate does your child have?		
Not affected	22	14.1
Incomplete	48	30.8
Complete	86	55.1
Was the cleft diagnosed during pregnancy?		
No	112	71.8
Yes	44	28.2
Does the child have any associated anomalies?		
No	102	65.4
I do not know	14	9
Yes	40	25.6
If the child had associated anomalies, what are they? n = 129		
Congenital cardiac anomalies	18	29
Hearing disability	20	32.3
Cognitive disability	6	9.7
Mental disability	3	4.8
Other problems	10	16.1
Speech difficulty	2	3.2
Dental problems	2	3.2
Esophageal anomalies	1	1.6
What is/are your sources of information about CLP?		
Internet	46	29.5
Social media	31	19.9
Hospital	72	46.2
Family member	9	5.8
Causal meeting	1	0.6
When was the first time you heard about CLP?		
During the pregnancy	77	49.4
After having a child with CL/C	79	50.6

CLP = cleft lip and/or cleft palate.

**Table 3:** Descriptive analysis of parental knowledge of CLP.

	Frequency	Percentage
What is a cleft?		
Opening of lips only	22	14.1
Opening on the lips and hard palate	103	66
Opening on the hard palate only	29	18.6
I do not know	2	1.3
Could CLP be syndromic or not?		
No	65	41.7
I do not know	69	44.2
Yes	22	14.1
Could CLP have other associated anomalies or not?		
No	15	9.6
I do not know	41	26.3
Yes	100	64.1
Can CLP be diagnosed before the child is born?		
No	7	4.5
I do not know	33	21.2
Yes	116	74.4
Can we prevent CLP?		
No	48	30.8
I do not know	88	56.4
Yes	20	12.8
Which of the following is a known CLP risk factor? n = 281		
Chemical substance exposure during pregnancy	58	49.2
Consanguineous marriage	47	39.8
Nicotine exposure	64	54.2
Medicines like phenytoin, valproic acid, trimethadione	80	67.8
Genetic	32	27.1
Maternal illness	81	68.6
Stress	73	61.9
Inadequate Intake of Folic acid during pregnancy	44	37.3
Does the management require a multidisciplinary approach?		
No	6	3.8
I do not know	8	5.1
Yes	142	91
Who are the participants of the team?		
Prosthodontist	135	86.5
Orthodontist	135	86.5
Social workers	129	82.7
Otolaryngologist	150	96.2
Pedo-dentist	135	86.5
Speech therapist	129	82.7
Geneticist	129	82.7
Plastic/craniofacial surgeon	149	95.5
Feeding specialist	149	95.5
Nurse coordinator	149	95.5
Is surgery a known treatment for CLP?		
No	3	1.9
Yes	153	98.1
Is a Prosthesis a known treatment for CLP?		
No	66	42.3
I do not know	61	39.1
Yes	29	18.6
Are medications part of treatments for the CLP?		
No	90	57.7
I do not know	41	26.3
Yes	25	16
Do you think that the management of CLP is done at different stages in the child’s life?		
No	3	1.9
I do not know	9	5.8
Yes	144	92.3
What is the correct age for doing the mentioned procedure [Cleft lip repair]		
2 wk after birth till 6 mo after birth	33	21.2
3–6 mo	106	67.9
9–18 mo	17	10.9
What is the correct age for doing the mentioned procedure [Palatoplasty]		
2 wk after birth till 6 mo after birth	10	6.4
3–6 mo	22	14.1
9–18 mo	124	79.5
What is the correct age for doing the mentioned procedure [Naso alveolar Molding (NAM)]		
2 wk after birth till 6 mo after birth	43	27.6
3–6 mo	36	23.1
9–18 mo	77	49.4
What are the possible complications for (untreated) CLP children?		
Speech difficulties	121	77.6
Recurrent middle ear infection and hearing loss	83	53.2
Facial deformity	101	64.7
Psychological distress,	56	35.9
Abnormal dental development,	4	2.6
Feeding difficulties	120	76.9
Breathing problems	1	0.6

CLP = cleft lip and/or cleft palate.

When asked about known risk factors, more than half of parents correctly linked CLP to nicotine use (54.2%), teratogenic medications (67.8%), maternal illness (68.6%), and stress (61.9%). Still, fewer recognized the role of consanguinity (39.8%) or inadequate folic acid intake (37.3%), and only a quarter (27.1%) identified genetics as a factor. Encouragingly, most parents (91%) were aware that CLP management requires a multidisciplinary team. Many correctly identified key team members, such as otolaryngologists (96.2%), surgeons (95.5%), feeding specialists (95.5%), and others, including prosthodontists, speech therapists, and nurse coordinators (all above 82%). Surgical intervention was widely recognized as the primary treatment (98.1%), though fewer parents were aware of supportive interventions. Only 18.6% recognized prosthetics as a treatment option, and just 16% identified medications as part of the management plan, suggesting the need for broader education on the full scope of care beyond surgery. In the continuation of Table 3, most parents (92.3%) correctly recognized that CLP management occurs in multiple stages throughout the child’s development. Regarding timing of procedures, 67.9% of respondents accurately identified 3–6 months of age as the appropriate window for cleft lip repair, and 79.5% knew that Palatoplasty is typically performed between 9 and 18 months. However, only 27.6% selected the correct early-age window for NAM, suggesting limited awareness of presurgical interventions used during infancy.

When asked about complications associated with untreated CLP, most parents demonstrated strong awareness of key risks. The majority recognized speech difficulties (77.6%), feeding problems (76.9%), and facial deformity (64.7%) as potential consequences. Over half (53.2%) were aware of the link with recurrent ear infections and hearing loss, and just over a third (35.9%) identified psychological distress. However, fewer parents recognized abnormal dental development (2.6%) and breathing problems (0.6%) as possible outcomes, suggesting that while general awareness of major complications is high, knowledge of more specific or less visible risks remains limited.

Table 4 displays the descriptive analysis of parental practices regarding CLP. The vast majority of parents (83.3%) sought medical attention immediately upon noticing their child’s condition. However, 5.1% reported delaying care, and 11.5% indicated they never sought medical attention at all. Among those who delayed, half waited over a year before pursuing care, underscoring a concerning delay in early intervention for some families. In terms of feeding practices, formula feeding was the most common approach (67.9%), with only 6.4% relying exclusively on breastfeeding. A quarter of respondents (25.6%) reported using both methods. Most parents (87.9%) adopted appropriate feeding positions, with 46.2% using a semi-upright 45-degree position and 41.7% feeding their child fully upright, both of which help reduce aspiration risk. Only 12.2% used the supine position, which is generally discouraged. The majority of families (81.4%) used regular formula, while 18.6% opted for thickened formula to aid feeding. Encouragingly, 67.3% of parents reported using specialized feeders or prostheses to support adequate nutrition, reflecting good adherence to clinical feeding recommendations for CLP management.

**Table 4:** Descriptive analysis of parental practices regarding CLP-affected children.

	Frequency	Percentage
When did you seek medical attention?		
When we noticed immediately	130	83.3
We waited a while	8	5.1
We did not seek medical attention	18	11.5
If you chose “waited a while” for the previous question, for how long have you waited?		
≤2 mo	1	12.5
6–12 mo	3	37.5
>1 yr	4	50
What type of feeding does your child mainly depend on? (If applicable)		
Formula feeding	106	67.9
Breastfeeding	10	6.4
Both Formula and Breastfeeding	40	25.6
Which position do you use while feeding your child?		
Supine	19	12.2
45°	72	46.2
Upright (90°)	65	41.7
What type of formula do you use for your child with CLP?		
Regular formula	127	81.4
Thick formula	29	18.6
Do you use special feeders/prostheses while feeding your child with CLP?		
No	51	32.7
Yes	105	67.3

CLP = cleft lip and/or cleft palate.

Table 5 explores parents’ perceptions of healthcare services for children with CLP. Just under half of the parents (45.5%) felt they had been adequately informed about CLP. In comparison, a substantial portion remained neutral (29.5%) or expressed disagreement to some extent (25%), indicating room for improvement in public and provider-driven education. Most families (59.6%) reported having to travel for appointments, with nearly 55% describing the specialized care center as either “quite far” or “very far.” Travel times were often burdensome, with 43.6% of parents reporting that it takes over an hour to reach their child’s follow-up center. Despite these challenges, over half of the parents (51.9%) described obtaining school leave for medical appointments as “easy,” though 24.4% found it “quite” or “very” difficult. Work obligations also interfered with care – about 31% had missed at least 1 appointment due to job responsibilities. Fewer parents reported missing appointments due to school conflicts (12.8%) or the lack of a caregiver for other children (16%). Delays in access to medical appointments were also evident in reported waiting times: over half of respondents (52.6%) waited more than 7 days for physician appointments, and 44.2% said the interval between consultations exceeded 7 weeks. These findings suggest that while families are motivated and engaged, logistical barriers and scheduling challenges remain significant concerns in ongoing CLP care among Saudi families.

**Table 5:** Descriptive analysis of parental perceptions about their CLP children’s medical services and appointments.

	Frequency	Percentage
Do you think you have been provided with enough information about CLP from your doctor/the community? mean (SD)-Agreement		3.68 (1.23)
Completely disagree	29	18.6
Disagree	10	6.4
Neutral	46	29.5
Agree	24	15.4
Completely agree	47	30.1
Do you travel for your appointments?		
No	63	40.4
Yes	93	59.6
How far is the specialized care center that your child with CLP follows up at from your residence?		
Very Near	4	2.6
Near	36	23.1
Slightly far away	30	19.2
Quiet Far	44	28.2
Very far	42	26.9
How much time do you need to get to the specialized care center where your child with CLP follows up?		
<15 min	4	2.6
15–30 min	25	16
40–60 min	32	20.5
60 min	27	17.3
>1 h	68	43.6
How easy/difficult is it to obtain a school leave for your child’s medical appointments?		
Very easy	23	14.7
Easy	81	51.9
Slightly difficult	14	9
Quiet difficult	33	21.2
Very difficult	5	3.2
How many medical appointments have you missed because of your work?		
None	108	69.2
One	13	8.3
Two	12	7.7
Three	6	3.8
Four or more	17	10.9
How many medical appointments have you missed because of your child’s school?		
None	136	87.2
One	8	5.1
Two	3	1.9
Three	2	1.3
Four or more	7	4.5
How many medical appointments have you missed because you could not find a caregiver for your other children?		
None	131	84
One	8	5.1
Two	5	3.2
Three	5	3.2
Four or more	7	4.5
What is the average waiting time for a physician’s appointment?		
1–6 d	25	16
1–2 wk	21	13.5
3–6 wk	28	17.9
≥7 wk	82	52.6
How long is the interval between 2 consecutive consultations?		
At the patient’s convenience	25	16
1–3 wk	22	14.1
4–6 wk	40	25.6
≥7 wk	69	44.2

CLP = cleft lip and/or cleft palate.

Table 6 presents summary scores reflecting parents’ overall knowledge and perceived access to CLP care. The mean knowledge score was 23.12 (SD = 5.2) out of a possible 28 points, indicating that, on average, parents demonstrated a high level of understanding regarding CLP-related concepts, treatment procedures, and associated complications. In contrast, the mean perceived difficulty score for accessing CLP-treating physicians was 17.96 (SD = 4.84) out of 36, suggesting that many parents experienced a moderate level of challenge in securing timely or convenient access to specialist care. This discrepancy highlights a potential gap between what families know and their ability to act on that knowledge due to access-related barriers.

**Table 6:** Descriptive analysis of the parents’ overall perceptions and knowledge about CLP.

	Maximum possible score	Mean (SD)-score
Total CLP knowledge score (0–28 points)	0–28 points	23.12 (5.2)
Mean perceived difficulty having access to CLP-treating physicians	0–36 points	17.96 (4.84)

CLP = cleft lip and/or cleft palate.

Table 7 presents the bivariate correlations between parents’ CLP knowledge and other measured perceptions. The resulting findings suggested that parents’ total knowledge about CLP was moderately and significantly correlated with their household SES index (r = .433, *P* < .01). The SES index in this study is a composite, factor-based score that reflects parents’ educational levels, household income, and self-reported economic class. This positive association suggests that families with higher social status, those who are more educated and financially stable, tend to have better access to health-related information and greater awareness of their child’s condition.

**Table 7:** Bivariate Pearson correlations between the study-measured concepts.

	Knowledge score	SES Index	DIFF
Total CLP Knowledge score (0–28 points)	1.000	.433**	−0.079
Household Socioeconomic (SES) Index	.433**	1.000	−0.113

** Correlation is significant at the 0.01 level (2-tailed). *. Correlation is significant at the 0.05 level (2-tailed).

CLP = Cleft lip and/or cleft palate.

Table 8 presents a multivariable linear regression model identifying the predictors of parental knowledge regarding CLP. Household SES emerged as a strong and statistically significant predictor (B = 1.55, *P* < .001), indicating that for every unit increase in SES, the parents’ CLP knowledge scores increased by 1.55 points on average. This finding reinforces the role of the SES of families in shaping access to health information and CLP knowledge gain. Additionally, feeding position was a significant predictor (B = 1.20, *P* = .010), with parents using recommended positions (e.g., upright) demonstrating better knowledge scores. Although the number of missed medical appointments due to work showed a negative trend with knowledge (B = –0.43, *P* = .062), this association did not reach statistical significance. Likewise, children’s age was not a significant predictor of parents’ knowledge of CLP. Overall, the model suggests that both socioeconomic factors and lived caregiving experiences (e.g., feeding and observing complications) are key contributors to parental understanding of CLP. The study’s other measured predictor independent variables did not converge significantly on the parental knowledge score of CLP; as such, these factors were dismissed during iterative multivariable models.

**Table 8:** Multivariable linear regression analysis of parental Knowledge score on CLP.

	Unstandardized beta coefficient	95% CI for beta coefficient		
		Lower	Upper	*P*-value
(Constant)	12.968	10.300	15.637	<.001
Age of the child	0.028	−0.135	0.192	.733
Household Socioeconomic (SES) Index	1.548	0.805	2.291	<.001
Which position do you use while feeding your child Upright	1.203	0.290	2.116	.010
How many medical appointments have you missed because of your work?	−0.429	−0.880	0.022	.062

Dependent variable: parents’ means CLP total knowledge score.

CLP = cleft lip and/or cleft palate.

## 4. Discussion

CLP remains one of the most prevalent congenital anomalies worldwide, necessitating coordinated, multidisciplinary management from infancy through adolescence.^[2,13]^ Despite its global burden, there remains a significant lack of region-specific data addressing how parental socioeconomic factors, health literacy, and healthcare accessibility influence CLP understanding and management, particularly within underrepresented regions such as the Middle East, the Arab Gulf, and South Asia.^[5,14,15]^ Previous studies have established that social determinants of health critically affect health-seeking behaviors, timely intervention, and continuity of care in congenital conditions.^[5,16]^ This study aimed to assess parental knowledge, practices, and access to CLP care within Saudi Arabia, a context characterized by unique cultural, economic, and geographic factors influencing cleft management.^[17,18]^ Consistent with findings from both high and middle-income settings, higher parental educational attainment correlated positively with improved health literacy and greater adherence to recommended care regimens for children with CLP.^[5,16]^ This highlights the urgent need for targeted educational interventions tailored to diverse literacy levels to ensure equitable dissemination of information and facilitate parental engagement in treatment planning.

Genetic predispositions, including consanguinity, which remains prevalent in Middle Eastern populations, have been repeatedly associated with CLP incidence, emphasizing the importance of region-specific public health messaging and genetic counseling.^[16,19]^ Notably, our findings reveal a substantial underrecognition among parents of modifiable risk factors such as consanguineous marriage, inadequate maternal nutrition, and insufficient folic acid supplementation. These gaps represent missed opportunities for preventive education and highlight the imperative for culturally sensitive reproductive health campaigns. Public health initiatives integrating genetic counseling with periconceptional folic acid promotion could reduce CLP incidence in this population.^[9,11]^

Prenatal diagnosis of CLP remains suboptimal throughout much of the Arab Gulf, likely attributable to limitations in obstetric imaging quality, provider training, and service accessibility.^[20,21]^ This deficiency results in delayed parental preparedness and postponed intervention, with many families first learning of the diagnosis postnatally. Enhancing prenatal screening capabilities and healthcare provider training may improve early detection, enabling timely counseling and intervention planning.

A considerable proportion of children with CLP present with concomitant anomalies such as hearing impairments or cardiac defects, complicating clinical management and reinforcing the need for comprehensive evaluation and parental education.^[3,22]^ While awareness of the necessity for surgical correction and multidisciplinary follow-up was generally high, significant knowledge gaps persisted regarding the syndromic nature of CLP and its associated dental and psychosocial complications. These findings align with prior regional and international studies that report ongoing public misconceptions concerning the broader implications of CLP beyond surgical repair.^[6,9,11]^ Addressing these deficits requires comprehensive educational initiatives encompassing developmental, nutritional, and psychosocial dimensions of CLP care.

Parental practices related to feeding demonstrated encouraging adherence to recommended techniques such as upright positioning and the use of specialized feeding bottles. Nonetheless, exclusive breastfeeding rates remained low, consistent with existing literature highlighting feeding challenges in infants with CLP.^[15,22]^ Feeding difficulties continue to impose a substantial burden on caregivers and may contribute to early weaning and suboptimal nutritional outcomes. Accordingly, integrating lactation support and feeding counseling within early cleft care protocols is essential to enhance nutritional status and caregiver confidence.

Geographic and logistical barriers to accessing specialized cleft care emerged as significant obstacles, corroborating findings from other Middle Eastern and resource-limited contexts.^[18,19]^ Extended travel distances, protracted intervals between appointments, and work-related conflicts were frequently reported, resulting in delayed or missed clinical encounters. These structural challenges have direct consequences on the timing and effectiveness of interventions, particularly considering the critical windows for optimal surgical timing and speech development.^[9,20]^ Expanding decentralized cleft care centers and implementing telemedicine platforms may mitigate these barriers and improve continuity of care.

Healthcare providers remained the primary source of CLP-related information despite widespread internet access, highlighting their pivotal role in shaping parental understanding and expectations. However, nearly half of the surveyed parents reported inadequate information regarding their child’s condition, suggesting a pressing need for structured, consistent communication strategies during clinical consultations.^[11,12]^ Development of culturally appropriate educational materials, interactive counseling sessions, and repeated clear messaging may help bridge knowledge gaps and empower caregivers to participate actively in their child’s care.

Parental confidence in navigating the healthcare system, understanding treatment plans, and recognizing therapeutic progress closely correlates with the clarity and consistency of information provided. Inconsistent messaging, fragmented referrals, and perceived inaccessibility can undermine caregiver trust and adherence. Prior research has demonstrated that psychological distress and confusion surrounding CLP management negatively impact compliance and overall family well-being.^[10,12]^ Our findings reinforce the necessity to enhance counseling services, improve interdisciplinary coordination, and establish clear, structured care pathways as priorities in cleft care programs.

The present study has several limitations. To begin, recall bias can affect self-reported data, subsequently introducing the possibility of selection bias. As the study was performed using an online survey format, participants were limited to those with internet access, which may exhibit selection bias. The study was conducted in a single country and was limited to participants who responded to the survey, which may not fully represent all parents of children with CLP. There is a possibility of knowledge bias, as parents of children with CLP may already have a particular interest in CLP, leading to an overestimation of the level of knowledge in the broader population. As the online survey was initially formulated in English and then translated into Arabic, differences in interpretation may affect responses. Confounding factors such as the severity of the disease, the presence of other comorbidities along with syndromic CLP, and subsequent parental knowledge of such medical conditions are not controlled for in this study. However, despite the aforementioned limitations, this study provides key insight into parental perspectives on CLP and recognizes concerns for future research and intervention.

## 5. Conclusion

The present study provides insights into the knowledge, practices, and perceived challenges faced by Saudi parents caring for children with CLP. Findings highlight a proficient level of parental knowledge about CLP, particularly regarding the necessity of multidisciplinary management and the importance of surgical interventions. However, considerable gaps remain, particularly in awareness of preventive measures, syndromic associations, and less visible complications. Parental practices indicate strong adherence to recommended feeding methods, yet challenges persist in early medical intervention due to delayed care-seeking behavior among some families. Accessibility issues, including lengthy travel times and prolonged intervals between medical appointments, exhibited substantial barriers affecting ongoing healthcare engagement. Furthermore, SES influenced parental knowledge, underscoring the need for targeted educational initiatives and improved healthcare accessibility, particularly for lower socioeconomic groups. This study emphasizes the necessity of implementing systematic improvements in public health education, early diagnosis, and support services to bridge knowledge gaps and improve care outcomes for CLP-affected children in Saudi Arabia.

## Acknowledgments

The authors express their thanks and gratitude to the Deanship of Scientific Research at Princess Nourah bint Abdulrahman University for funding this research.

## Author contributions

**Conceptualization:** Sari M. Rabah, Alya AlZabin, Ebtesam Almajed.

**Data curation:** Heba Jafar Sabbagh, Alya AlZabin, Ebtesam Almajed, Razan Albrahim, Reema Aldawish, Lara Alyahiwi, Rand Alshabnan, Narmin Mohamed Helal.

**Formal analysis:** Heba Jafar Sabbagh, Alya AlZabin.

**Funding acquisition:** Sari M. Rabah, Alya AlZabin, Ebtesam Almajed.

**Investigation:** Alya AlZabin.

**Methodology:** Sari M. Rabah, Heba Jafar Sabbagh, Alya AlZabin, Razan Albrahim, Reema Aldawish, Lara Alyahiwi.

**Project administration:** Sari M. Rabah, Alya AlZabin, Ebtesam Almajed.

**Resources:** Alya AlZabin.

**Supervision:** Sari M. Rabah.

**Validation:** Alya AlZabin, Ebtesam Almajed.

**Visualization:** Alya AlZabin.

**Writing – original draft:** Alya AlZabin, Ebtesam Almajed, Razan Albrahim, Reema Aldawish, Lara Alyahiwi, Rand Alshabnan.

**Writing – review & editing:** Sari M. Rabah, Heba Jafar Sabbagh, Alya AlZabin, Ebtesam Almajed, Razan Albrahim, Reema Aldawish, Lara Alyahiwi, Rand Alshabnan, Narmin Mohamed Helal.
